# Beetroot as a functional food with huge health benefits: Antioxidant, antitumor, physical function, and chronic metabolomics activity

**DOI:** 10.1002/fsn3.2577

**Published:** 2021-09-09

**Authors:** Liping Chen, Yuankang Zhu, Zijing Hu, Shengjie Wu, Chengtao Jin

**Affiliations:** ^1^ Department of Pharmacy School of Medicine Sir Run Run Shaw Hospital Zhejiang University Hangzhou China; ^2^ College of Second Clinical Medical Wenzhou Medical University Wenzhou China; ^3^ Chemical Biology Research Center College of Pharmaceutical Sciences Wenzhou Medical University Wenzhou China

**Keywords:** beetroot, betanin, biological activity, health benefits, nitrate

## Abstract

Previously, beetroot is mainly consumed as a food additive. In recent years, the beetroot, especially the betalains (betanin) and nitrates it contains, now has received increasing attention for their effective biological activity. Betalains have been proven to eliminate oxidative and nitrative stress by scavenging DPPH, preventing DNA damage, and reducing LDL. It also has been found to exert antitumor activity by inhibiting cell proliferation, angiogenesis, inducing cell apoptosis, and autophagy. In some chronic diseases, nitrate is the main component for lowing blood lipids, glucose, and pressure, while its role in treating hypertension and hyperglycemia has not been clearly stated. Moreover, the intake of nitrate‐rich beetroot could enhance athletic performance and attenuate muscle soreness in certain types of exercise. The objective of this review is to provide sufficient evidence for the clarification of health benefits of beetroot, especially in the aspect of biooxidation, neoplastic diseases, some chronic diseases, and energy supplementation.

## Graphical abstract

A review of the latest research on the biological activities of nutritional composition in beetroot including antioxidant, antitumor, physical function, chronic metabolomics activity, and other functions.

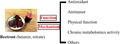

## 1 INTRODUCTION

**FIGURE 1:**
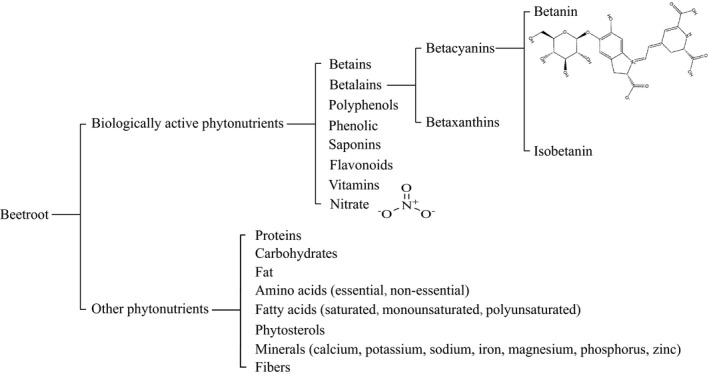
The main phytonutrients in beetroot

Nowadays, a large number of consumers have a strong preference for the “functional foods” for the improvement of their eating diet and maintenance of their health (Jeffery, 2005; Shashirekha et al., 2015). Accordingly, fruits and vegetables are important parts of the healthy diet with the capacity of preventing several diseases (Jeffery, 2005; Shashirekha et al., 2015). In recent years, the beetroot (*Beta vulgaris L*.) has become popular as a potential “functional food” within this context (Frank et al., 2005). In spite of the fact that the beetroot has long been used as a traditional cuisines in Europe, the understanding of the applied value is very limited. Today, with the development of preclinical trials, consumers have increased knowledge about the biological activity of beetroot. The beetroot is now widely cultivated to meet the increase in demand (Maity et al., 2016). In addition to being known as fresh vegetables, or as food additives in cattle products, beverages, candies, and dairy products (Georgiev et al., 2010; Vieira Teixeira da Silva et al., 2019), beetroot has also been found to possess the potential of treating and preventing multiple diseases. According to the database displays in the US Department of Agriculture Agricultural Research Service, beetroot is not only rich in proteins (1.68 g), carbohydrates (9.96 g), fat (0.18 g), amino acids (1.216 g), fatty acids (0.119 g), phytosterols (0.025 g), minerals (0.483 g), and fibers (2 g) per 100 g of wet weight, but also contains a lot of biologically active phytonutrients (Figure 1). The contents of vitamins and nitrate are 4.805 mg and 25 mg per 100 g of wet weight respectively. 3.976 g/100 g of betalains (2.075 g/100 g of betacyanins and 1.901 g/100 g of betaxanthins) and 0.1899 g/100 g of phenolic are produced in dry extract of beetroot. These bioactive phytonutrients have been proven as key ingredients for the treatment of some chronic diseases including cardiovascular and cerebrovascular diseases, cancer, diabetes, and chronic respiratory diseases. For instance, betalains (mainly betanin) are the effective antioxidant extracted from beetroot. Several lines of evidence have shown that betalains might reduce the risk of some cancers, cardiovascular and cerebrovascular diseases, liver and kidney damage (Kavitha et al., 2013). The nitrate also has great nutritional value in beetroot. A lot of consumers tend to take oral fresh beetroot juice to supplement nitrate and thus to positively affect the physiological reaction, reduce the risk of cardiovascular, and cerebrovascular diseases (Webb et al., 2008). The beetroot has now been widely used as a common vegetable for athletes to replenish energy.

Although a wide variety of researches have highlighted the bioactivity of beetroot in antioxidant, anti‐inflammatory, antitumor, hepato‐protective, cognitive improvement, blood pressure regulation, the value of its application is limited to natural colorant and functional beverage, overall. The main reason for such a situation is that the current researches on beetroot are still mainly concentrated at the cellular and animal level, lacking scientific, and credible clinical data. In spite of this, as a convenient, safe, and edible vegetable, the clinical verification of beetroot for health promotion is possible only a matter of time. In this article, we aim to review the related biological activity of the main active ingredients, hoping to provide some references for the future beetroot application in food manufacturing and medical care.

## 2 BIOLOGICAL ACTIVITY

### 2.1 Antioxidant activity

Since the emergence of processed foods, the demand for that has never decreased. It has also become increasingly common to add a certain amount of antioxidants to processed foods. Especially today, it is more urgent to make full use of antioxidants to extend shelf life and ensure food quality for many processed foods that are rich in high protein and fat (Ranawana et al., 2018). Although many synthetic antioxidants such as butylated hydroxyanisole (BHA) and butylated hydroxytoluene (BHT) are widely used in the world, natural antioxidants are highly sought after at home and abroad for their possible greater safety and health‐promoting effect (Liu et al., 2015; Wettasinghe et al., 2002). Polyphenols and anthocyanin both belong to natural antioxidants, even exhibiting much stronger antioxidant capacity than BHA and BHT (Guitard et al., 2016; Narayan et al., 1999). In addition, the antioxidant capacity of betanin, the major betalains in beetroot, is gradually receiving greater attention in response to public health demands. It is one of the few red dyes approved for food manufacturing known as E162 (Kapadia et al., 2011). Its antioxidant ability is also constantly being researched and reported. Some studies have even shown that the free radical scavenging ability of betanin in beetroot is nearly twice as high as some anthocyanins under the circumstance of pH >4 (Borkowski et al., 2005). The free radical scavenging ability and high antioxidant activity of betanin are linked to the presence of phenolic hydroxy groups in the structure (Costa et al., 2017). Previous reports have also indicated that the antioxidant activity of betanin is associated with the rich unsaturated bonds on the benzene ring (Butera et al., 2002). As for the specific active functional groups, further research and confirmation are needed.

Apart from having betanin, beetroot also contains a considerable amount of polyphenols and phenolic, a small quantity of vitamin C and vitamin E, which have been proved with the great antioxidant ability (Apak et al., 2004). Since betanin is the main active ingredient in beetroot, the researches on the antioxidant activity of beetroot are mainly concentrated upon betanin, and a small number of studies focus on the antioxidant activity of polyphenols (Koss‐Mikołajczyk et al., 2019). Studies have indicated the content of total polyphenols in methanol extracts of pulp waste from beetroot ranged from 67 to 110 mg TAE (tannic acid equivalents)/100 g sample, which was higher than ethanol extract and aqueous extract (Wettasinghe et al., 2002).

In spite of the fact that beetroot has been widely used as a colorant in food and provides antioxidant protection to a certain extent simultaneously, it is relatively rare for the main purpose of serving as antioxidant rather than food colorant to add into processed food. Research on how to maximize the antioxidant potency of beetroot is also in its infancy. Vikas Kumar et al. once used the beetroot pomace extract to develop ginger sugar, hoping to compensate for the defects in the antioxidant capacity damage of ginger‐based products caused by blanching technology (Kumar et al., 2018). It was found that optimum conditions for optimal oxidation titer of the ginger product were 7.81 min of blanching time and 9.24% concentration with 0.905 desirabilities of beetroot pomace extract. In this case, its antioxidant activity could reach to 88.44% and the content of phenolics and betalains was high as 273.29 mg/100 g and 31.54 mg/100 g, respectively. Another study found that in sponge cakes, the combination of beetroot and chocolate further improved the polyphenol profiles, oxidative stability, and extended its shelf life (Ranawana et al., 2018). It is mainly due to the fact that chocolate is more effective in retarding lipid oxidation than beetroot. This study is hopeful to provide a reference for the future combination of beetroot and these substances with good anti‐lipid oxidation capacity. Beyond that, the different processing type of beetroot also has a great influence on its antioxidant capacity. Burcu Guldiken et al. compared the effects of different processing methods including fresh samples, boiled, pickled, oven‐dried, pureed, jam‐processed, and juice‐processed on antioxidant activity (Guldiken et al., 2016). It was found that fresh, dried, and pureed beetroot exhibited the highest TP (total phenolic), TF (total flavonoid), and TAC (total antioxidant capacities) values. In recent years, it was then reported that freezing with liquid nitrogen resulted in greatest increase in TP of beetroot (Dalmau et al., 2019). Anne Porto Dalla Costa et al. pointed out that beetroot waste flour was a convenient processing method. It was advised to dry at 70 ℃ to obtain the maximum amount of betalains content and the best antioxidant activity (Costa et al., 2017).

**FIGURE 2:**
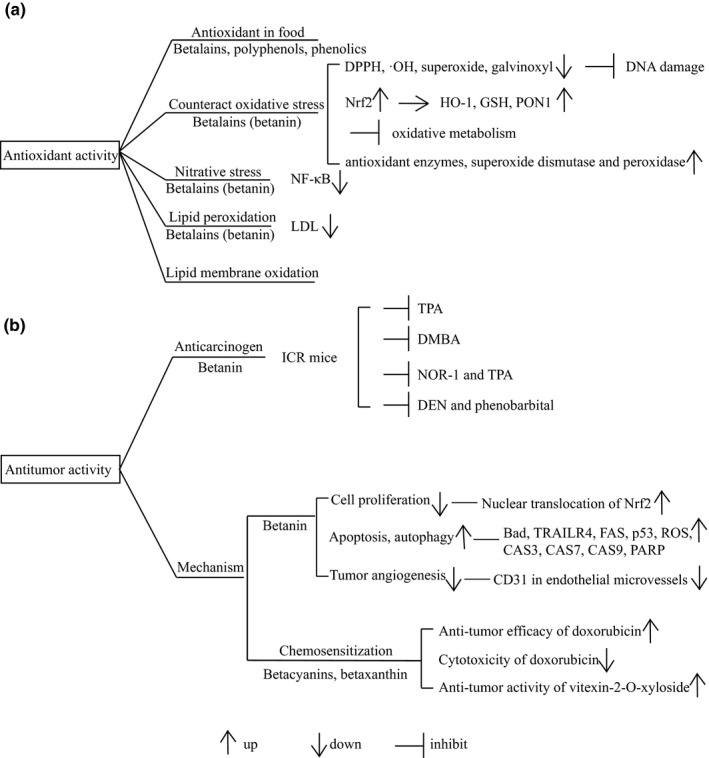
Diagram of antioxidant activity (a) and antitumor activity (b) of beetroot. DPPH, 2,2diphenyl‐1‐picrylhydrazyl; Nrf2, nuclear factor (erythroid‐derived 2)‐like 2; HO‐1, Heme oxygenase; GSH, glutathione; PON1, paraoxonase 1; NF‐κB, nuclear factor‐kappa B; LDL, low‐density lipoprotein; TPA, 12‐O‐tetradecanoylphorbol‐13‐acetate; DMBA, 7,12‐dimethylbenz‐(a)anthracene; NOR‐1, (±)‐(E)‐4‐methyl‐2‐[(E)‐hydroxyamino]‐5‐nitro‐6‐methoxy‐3‐hexanamide; DEN, N‐nitrosodiethylamine; TRAILR4, tumor necrosis factor‐related apoptosis‐inducing ligand 4; FAS, factor associated suicide; ROS, reactive oxygen species; CAS3, caspase‐3; CAS7, caspase‐7; CAS9, caspase‐9; PARP, poly ADP‐ribose polymerase

In addition to the above‐mentioned function of using beetroot as an antioxidant in food, more researches currently focus on the maintenance of balance by beetroot to counteract oxidative stress, thereby preventing and treating human diseases. Betanin in beetroot was reported both to be a free radical scavenger and an inducer of antioxidant defense mechanisms in cultured cells (Esatbeyoglu et al., 2014). It could scavenge DPPH (2,2diphenyl‐1‐picrylhydrazyl), hydroxyl‐radicals, superoxide, and galvinoxyl in a concentration‐dependent manner and prevent DNA damage induced by hydrogen peroxide. The study revealed that Nrf2 (nuclear factor (erythroid‐derived 2)‐like 2) was the most likely involved signaling pathway that mediated the antioxidant activity. The transactivation of Nrf2 would then result in an increase of HO‐1 (HemeOxygenase), GSH (glutathione) proteins, and transactivation of PON1 (paraoxonase 1). As to whether such signal transduction pathway exists in the human body, there is no relevant proof yet. It was reported that under the gastrointestinal condition, betalains would remain stable without any significant loss in antioxidant properties, which further maintained the efficacy of beetroot for human disease treatment (Frank et al., 2005). To evaluate the antioxidant activity of beetroot, Malgorzata Kujawska et al. used a model of oxidative stress induced by two xenobiotics, N‐nitrosodiethylamine (NDEA), and carbon tetrachloride (CCl4) (Kujawska et al., 2009). The level of microsomal lipid peroxidation in rats pretreated with beetroot juice was found to decrease by 38%, while it was increased by several folds in rats administered with CCl4 alone. The reduced level of plasma protein carbonyls and diminished DNA damage in rats pretreated with beetroot juice before NDEA administration was similar to be detected. Additionally, pretreatment with beetroot juice caused a threefold increase in the activity of superoxide dismutase, reversing the downregulation of superoxide dismutase caused by oxidative stress model. This study has led the authors to believe that beetroot does possess promising antioxidant activity. As for the relevant antioxidants, it has not been elaborated and speculated that it might be the synergy of betalains and other compounds. Besides, betalains in beetroot have also been shown to inhibit oxidative metabolism of neutrophils in humans (Zielińska‐Przyjemska et al., 2009), enhance the activity of the antioxidant enzymes, superoxide dismutase, and peroxidase in irradiated mice liver, spleen, and kidney (Wroblewska et al., 2011). Apart from protecting cells from oxidative stress, it has been found that beetroot contributes to protecting cells from nitrative stress. In the study by Yasuko Sakihama et al., betanin in beetroot was proved to inhibit the nitration of peroxynitrate mediated by tyrosine in vitro (Sakihama et al., 2012). Previous research suggested that beetroot weakened the activation and nuclear translocation of NF‐κB to resist oxidative stress and nitrative stress induced by Gentamicin in rats (El Gamal et al., 2014). The similar protective effect with beetroot pretreatment was observed in rats with isoproterenol (ISP)‐induced myocardial injury (Raish et al., 2019). Moreover, it was indicated that the presence of betanin contributed to the reduction of LDL in humans that reflected lipid peroxidation (Tesoriere et al., 2004). It was responsible for the postponement of cumOOH (cumene hydroperoxide)‐induced oxidization (Tesoriere et al., 2005). The application of beetroot was also proven useful for the mitigation of lipid membrane oxidation caused by the change of H_2_O_2_‐mediated metmyoglobin and free iron in vitro (Kanner et al., 2001). The main antioxidant activity and related mechanisms described above were presented in Figure 2a.

### 2.2 Antitumor activity

Cancer remains one of the leading causes of human death in the world. According to reports, there were 17.5 million cancer patients existing in 2015 (Javadi, 2018). At present, conventional interventions such as chemotherapy, surgery, and radiation are often used to help cancer patients get rid of the disease (Dennert & Horneber, 2006). Although these interventions are available, the applications of which are also accompanied by some side effects (Dennert & Horneber, 2006). For safety concerns, there is a great need to look for effective and safe methods for cancer treatment. The surveys elsewhere have indicated that the forms of some alternative and complementary medicine might actually stand a chance against cancer disease with less adverse effects (Bozza et al., 2018; Trimborn et al., 2013). In fact, beetroot just belongs to such medicine. The reports suggested that beetroot could act as a chemopreventive medicine with the capacity of reducing the formation of multi‐organ tumors and exhibiting less toxicity. Here, we reviewed the antitumor activity of the active component in beetroot and hoped that the application of beetroot would indeed become a feasible measure for future cancer treatment.

Prior to 1996, Govind J. Kapadia et al. began to study the antitumor activity of beetroot (Kapadia et al., 1996). The results showed that the oral administration of betanin in beetroot contributed to the inhibition of skin and lung tumors in ICR mice pretreated with tumor promoter 12‐*O*‐tetradecanoylphorbol‐13‐acetate (TPA). Particularly in lung tumors, the reduction rate was up to 60%. It was the first report to illuminate the antitumor activity of beetroot using the animal model. With the development of research, the evidence emerging later also supports this finding. Govind J. Kapadia et al. once constructed three different tumor models in mice by using 7,12‐dimethylbenz(a)anthracene (DMBA), (±)‐(E)‐4‐methyl‐2‐[(E)‐hydroxyamino]‐5‐nitro‐6‐methoxy‐3‐hexanamide (NOR‐1) and TPA, *N*‐nitrosodiethylamine (DEN) and phenobarbital, respectively (Kapadia et al., 2003). During the experiment, they found that the betanin in beetroot significantly resisted the development of tumors caused by chemical carcinogens, suggesting that beetroot was a potent cancer chemopreventive agent. In addition, in HepG2 cells, treatment with beetroot component betanin resulted in the nuclear translocation of Nrf2 and then led to cell death (Krajka‐Kuźniak et al., 2013). More importantly, betanin‐enriched beetroot had no obvious effect toward normal cells while the cytotoxicity of beetroot characterized on cancer cells was found powerful (Nowacki et al., 2015). As the above results showed, beetroot was expected to be applied in the treatment of various tumors.

Up to now, researches have shown that inhibiting cell proliferation, promoting apoptosis, and autophagy are the main ways in which beetroot inhibits cancerous formation and development. For example, it was reported that beetroot (betalains and betaine) played a crucial role in reducing the number of esophageal papillomas induced by NMBA (N‐nitrosomethylbenzylamine) in rats (Lechner et al., 2010). The exact mechanism involved was found to be associated with the inhibition of cell proliferation and stimulation of cell apoptosis, which was measured by immunohistochemical staining of Ki‐67 and terminal deoxynucleotidyl transferase dUTP nick end‐labeling staining. Besides, Laëtitia Nowacki et al. found that the application of betanin‐enriched extract was involved in the restriction of cancer cell viability (Trimborn et al., 2013). The increase in the expression level of apoptosis‐related proteins (Bad, TRAILR4, FAS, p53) and alteration of the mitochondrial membrane potential was detected in MCF‐7 cells. Upon the beetroot treatment, it was also observed that autophagosome vesicles occurred in the MCF‐7 cells. In the study of other cells, the results existed likewise. The treatment of betanin separating from beetroot caused cleavage of procaspase‐3 and up‐regulation of ROS in CaCo‐2 cells (Zielińska‐Przyjemska et al., 2016). Based on these results, we conclude that beetroot exerts cancer‐preventive activity by inducing cell apoptosis. However, as to whether there exists some correlation between ROS and apoptosis, it remains to be studied. Beetroot (Betanin) could also induce cell apoptosis by activating the expression of caspase‐3, caspase‐7, caspase‐9, and PARP in human lung cancer cell lines (Zhang et al., 2013). In the study of rat model with CaCo‐2 cells, it was found that the combination of fermented beetroot juice (FBJ) and 2‐Amino‐1‐methyl‐6‐phenylimidazo[4,5‐b] pyridine (PhIP) abolished the cytotoxic and genotoxic effects induced by PhIP (Klewicka et al., 2012). Compared with PhIP‐induced model, the group fed with PhIP and FBJ would effectively reduce the content of malondialdehyde and MDA in vivo. The antioxidant status of serum was also increased by 26% in the combined group, which suggested that the beetroot intake, mainly the betalains, was likely to enhance its anti‐oxygenic ability and provide protection against the formation of a precancerous aberrant crypt. In addition to the above pathways, studies have found that beetroot (betanin) could exert antitumor effects by inhibiting angiogenesis. The research of beetroot chemopreventive activity was conducted in two different animal lung tumor models (Zhang et al., 2013). It was observed that the tumor multiplicity and load decreased in these two tumor models induced by Vinyl carbamate (VC)/benzo(a)pyrene (B(a)P) and beetroot. In the VC model, the tumor multiplicity and load were induced by 20% and 39%, respectively. In the model of B(a)P, the inhibition rate was up to 46% and 65%. Apart from the discovery of increased expression of caspase‐3, the number of CD31 in endothelial microvessels was found reduced after beetroot treatment, which suggested that cell apoptosis induction and angiogenesis inhibition were involved in the tumor inhibitory effects.

In addition to the above findings, the chemosensitization effects of beetroot in tumors also aroused great interest. Doxorubicin is an effective cytotoxic antitumor drug. It could be used clinically to treat various malignant tumor such as breast cancer, lung cancer, ovarian cancer, stomach cancer, and liver cancer (Cabeza et al., 2017; Dag et al., 2016; Marano et al., 2017). However, the use of doxorubicin is often accompanied by some side effects, significantly limiting its clinical application (Dag et al., 2016; Marano et al., 2017). At present, doxorubicin is mainly used in combination with other antitumor drugs. Studies have found that the antitumor efficacy was also improved by the combination of beetroot and doxorubicin. For instance, Sayantanee. Das et al. once studied the effects of co‐treatment with beetroot and doxorubicin on breast cancer cells MDA‐MB‐231 (Das et al., 2013, 2016). The results in this study showed that the ROS level and cell apoptosis both were increased with treatment of beetroot in MDA‐MB‐231 cells. Similarly, in the breast (MCF‐7), prostate (PC‐3), and pancreatic (PaCa) cells, the co‐administration of doxorubicin with beetroot exhibited synergistic effect (Kapadia et al., 2013). Although Govind J. Kapadia et al. had previously declared that beetroot (betanin) exhibited significantly lower cytotoxicity compared with doxorubicin toward normal cells (Kapadia et al., 2011), it must continue to further explore the differential toxicity using doxorubicin alone or in combination with beetroot. Furthermore, the similar combined effects have been observed in studies using BC (Betacyanins) and XVX (Vitexin‐2‐O‐xyloside). A study by E. S. Scarpa et al. suggested that the combined use of XVX and BC caused an enhanced antitumor activity concerning the increased expression of pro‐apoptotic Bax, and downregulation of anti‐apoptotic BIRC5 (Survivin) and pro‐survival CTNNB1 (β‐Catenin) in T24 bladder cancer cells (Scarpa et al., 2016). Unlike the previous research of doxorubicin and beetroot, it also pointed out the combination of XVX and BC had no cytotoxic effect toward normal cells NCTC 2544 (Scarpa et al., 2016). However, it is not enough to use only one normal cell line to clarify their combination has less toxicity. It is necessary to offer toxicity assessment on other normal cells and carry out studies as supporting evidence in vivo. In another study, the scholars were not limited to research the combined effects of XVX and BC, betaxanthin (BX) that generated due to the resonance of double bond in BC was also involved (Farabegoli et al., 2017). It was found that double combinations of XVX and BC, XVX and BX or triple combinations of XVX, BC and BX all caused an increase in cytotoxicity toward CaCo‐2 cells. This probably attributed to betalains for its ability to reduce the mRNA level of COX‐2 and IL‐8. For chronic lymphocytic leukemia (CLL) patients, it has begun to be gradually accepted that the application of beetroot–carrot juice provided a successful treatment for the complementary or alternative therapy combined with chlorambucil, a conventional leukemic treatment drug (Shakib et al., 2015). This effect was proved by the decrease of the number of leukocytes, lymphocytes in peripheral blood and improve the relevant biochemical parameters.

Through all of the above (Figure 2b), there is enough justification for the potential ability of beetroot used singly or combinedly for cancer treatment. However, due to the complexity of beetroot constituents, we cannot fully explain the contribution of a specific component to its antitumor activity yet. Some other components possibly also involved in minimizing the occurrence and stunting the development of cancer disease via other mechanisms. Perhaps, the different impacts of conventionally (CONV) and organically (ORG) produced beetroots on cancer cells were associated with these unknown compounds likewise (Kazimierczak et al., 2014). For instance, betaine also was proved as an antitumor component in beetroot despite its activity being lower than betanin (Lee et al., 2014). It was found that after exposure to betanin at a concentration of 200 μg/ml, the inhibition rate on HepG2 cells was 49%, while it was just 25% inhibition rate achieved when the concentration of betaine reached 800 μg/ml. Besides, because it is impossible to simulate the interaction between tumor and host by cell or animal experiments alone, the future research of beetroot should be more closely integrated with the human test model in vivo. Although beetroot rarely induces damage for its natural non‐toxic properties, it still needs to assess the probable interactions between beetroot and a conventional chemotherapy drug. It is guessed that the combined use with chemotherapy drug is likely to be the main aspect of future studies on the antitumor activity of beetroot.

### 2.3 Effect on blood lipids, glucose, and pressure

With the changes in current social living habits, diet, living environment, and other factors, hyperglycemia, high blood pressure, and high blood lipids have become nonnegligible chronic diseases that seriously threaten the human health (Anagnostis et al., 2015; Christofaro et al., 2014; Koskela et al., 2013). It is reported beetroot possesses the ability to alleviate these diseases and has the advantages of no side effects compared with synthetic drugs. Therefore, an in‐depth study of active ingredients in beetroot and its related pharmacological function in chronic metabolic disease is of great significance.

It is reported that the function of lowering blood pressure by beetroot is associated with an ingredient isolated from beetroot that is dietary nitrate. Under the action of bacterial anaerobes situated on the tongue, the dietary nitrate would be activated into nitrite, thus exhibiting vasoprotective effects (Webb et al., 2008). The interruption of such conversion results in no obvious vasoprotective activity (Webb et al., 2008). Besides, in hypercholesterolemic patients, the alterations of oral microbiome would also influence the change from dietary nitrate to nitrite and therefore affects their vascular function (Velmurugan et al., 2016). In chronic hypertension in pregnancy, it also speculated that the differences in efficacy of nitrate supplementation were related to differences in the oral microbiome (Ormesher et al., 2018). In addition to having proven that the nitrate is the decisive form of beetroot to regulate the vascular function, the awareness of the impact of beetroot on microvascular vasodilation and arterial stiffness related to the vasoprotective effects is gradually increasing. Ditte A. Hobbs et al. found that the intake of nitrate in beetroot led to an increased curve area for endothelium independent vasodilation and decreased diastolic blood pressure (BP) in humans (Hobbs et al., 2012, 2013). The results carried by Davi Vieira Teixeira da Silva's group indicated that the diastolic BP (5.2 mmHg), systolic BP (6.2 mmHg), and heart rate were decreased following the beetroot (nitrate) consumption in healthy subjects (Silva et al., 2016). The reduction of BP also was enhanced with beetroot supplementation following a moderate‐intensity aerobic exercise in obese individuals (Lima Bezerra et al., 2019). These findings have shown herein highlighted the testing of beetroot in vasoprotectiveness. Despite the above reports, the efficacy of beetroot in lowering blood pressure is affected by many other factors, such as age and sex (Coles & Clifton, 2012; Siervo et al., 2015). The presence of Glu298Asp polymorphism in the endothelial NO synthase (eNOS) gene also affects the postprandial blood pressure response to dietary nitrate‐rich beetroot bread (Hobbs et al., 2014). Recent research has raised different perspectives on the effects of beetroot diets on lowering blood pressure; that is, the consumption of raw beet juice (RBJ) and cooked beet (CB) has a difference in antihypertensive effects. The group of S. Asgary et al. once made a research and found that compared to CB, RBJ had a stronger antihypertensive effect in hypertensive subjects (Asgary et al., 2016). While regarding the reason for the different effect between CB and RBJ, it requires to be further studied. Apart from the above findings, the key problem regarding the beetroot is to clarify the activity on treating hypertension, despite it is generally considered playing a role in lowering blood pressure. Research once pointed that the normalization of mRNA expression in CTGF, MCP‐1, and MMP‐2 followed by beetroot treatment would lead to the improvement of the hypertension in rat model, and this effect was induced by nitrate rather than betaine (Bhaswant et al., 2017). Nevertheless, in the study of patients with hypertension, Bondonno et al. pointed out that there was no significant improvement of hypertension after beetroot intake (Bondonno et al., 2015). C. P. Kerley et al. stated briefly that the function of beetroot in antihypertensive was achieved only among those patients with uncontrolled hypertension (Kerley et al., 2017). The improvements in muscle microvascular function were also observed in never‐treated hypertensives (Zafeiridis et al., 2019). In addition, research provided evidence that nitrate‐rich beetroot had an acute effect on circulating immune cells and platelets in older adults owing to decreased blood monocyte‐platelet aggregates and reduced blood CD11b‐expressing granulocytes (Raubenheimer et al., 2017). In the subsequent study, therefore, the group determined to study whether the consumption of beetroot could reduce the risk of hypertension.

Hyperglycemia is another disease that the ingredients of betalains, polyphenols, and dietary nitrate in beetroot might have a great help for its treatment. It was found that with beetroot intake, the postprandial insulin response in the 0–60 min phase and the glucose response in the phase of 0–30 min were significantly downregulated in healthy volunteers (Wootton‐Beard et al., 2014). A result shown in *Biomedicine & Pharmacotherapy* further supported for the standpoint that beetroot exhibited beneficial effects in maintaining glucose homeostasis. Dhananjayan Indumathi et al. constructed a diabetic rat model induced by streptozotocin (STZ, 40 mg/kg b.w.) and nicotinamide (NA, 110 mg/kg b.w.) (Dhananjayan et al., 2017). After oral treatment with betanin, they found that followed by a significant increase of glycolytic enzyme (glucokinase and pyruvate kinase), glucose‐6‐phosphate dehydrogenase, and decrease in the activity of gluconeogenic enzymes (glucose‐6‐phosphatase and fructose‐1,6‐bisphosphatase), the levels of plasma glucose, insulin, glycosylated hemoglobin (HbA1c), and hemoglobin (Hb) were restored in diabetic rat. Unfortunately, however, because the current research on the anti‐hyperglycemia of beetroot is very limited, more research is urgently required.

Likewise, beetroot intake is benefit for hyperlipidemia treatment. The betalains in beetroot regulate peroxidation by scavenging lipid free radicals and then inhibiting peroxidase, nitrite‐induced oxidase, and human low‐density lipoprotein (Allegra et al., 2007). Wroblewska et al. revealed that the serum glucose levels, cecal atherosclerosis index, isovaleric acid concentration, and mice weight would significantly reduce in the hyperlipidemic model after feeding with beetroot (Sakihama et al., 2012). The inhibition of short‐chain fatty acids and serum total sterols was also observed in beetroot‐treated mice due to the increase in excretion of bile acids (Sakihama et al., 2012). Additionally, Zielinbska et al. carried an in vivo experiments with beetroot and found that following beetroot juice or beetroot slices intake, the oxidation metabolism of neutrophils was effectively inhibited in obese people, which thereby led to the reduction of body fat content (Wroblewska et al., 2011). These findings indicate that beetroot has potential application value for hyperlipidemia treatment.

**FIGURE 3:**
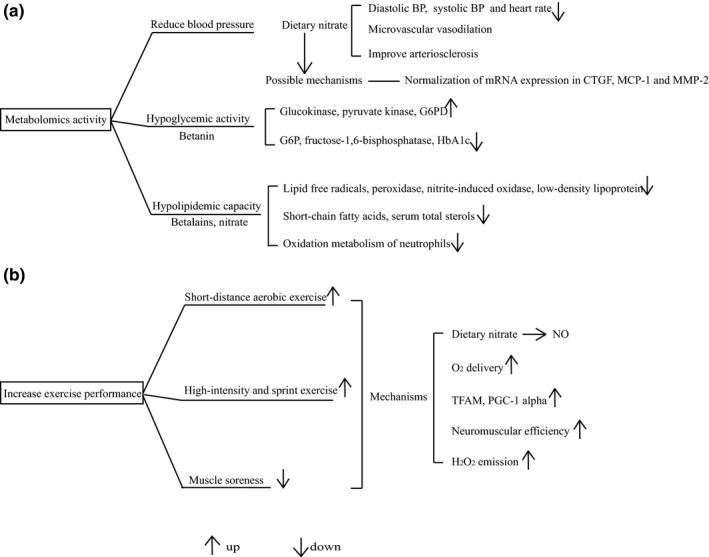
Diagram of metabolomics activity (a) and exercise performance increasing activity (b) of beetroot. BP, blood pressure; CTGF, connective tissue growth factor; MCP‐1, monocyte chemoattractant protein‐1; MMP‐2, matrix metculoproteinase‐2; G6PD, glucose‐6‐phosphate dehydrogenase; G6P, glucose‐6‐phosphatase; HbA1c, glycosylated hemoglobin; NO, nitric oxide; TFAM, mitochondrial transcription factor A; PGC‐1, peroxisome proliferator‐activated receptor gamma coactivator‐1 alpha

From the above research (Figure 3a) and the additional findings that nitrate in beetroot possesses the capacity of regulating flow‐mediated dilation (FMD) both in obese and normal‐weight people (Bakker et al., 2015; Joris & Mensink, 2013), we believe that beetroot underlies the potential of a low cost and effective approach for the treatment of chronic disease including hyperglycemia and hyperlipidemia as well as the reduction of blood pressure. At present, it is necessary to conduct more research in a larger population to determine its applied range.

### 2.4 Improve exercise performance

The interest in various nutrients for improving athletic exercise performance has increased in recent years (Berry et al., 2015). Due to the approved dietary nitrate on physical performance, nitrate‐rich beetroot thus attracts considerable attention. It is recognized that dietary nitrate decomposes into nitrite and subsequently converts to nitric oxide (NO) and other nitrogen‐active intermediates that affect the physical performance of athletic populations (Ferguson et al., 2013). However, NO may lead to the formation of N‐nitroso compounds (NOCs) following a secondary pathway, making it be cautious with beetroot intake (Berends et al., 2019). Up to now, many studies have been conducted on the impact of beetroot in the exercise performance of multifarious subjects.

In one study, for evaluating the possibility of supplementing beetroot juice to improve the athletic performance in short‐distance aerobic exercise, both the men and women athletes in national 500 m kayak team were chosen as research objects (Peeling et al., 2015). Peeling et al. then found that there were different increased degrees in exercise performance following the beetroot intake. However, it was found by Puype et al. that supplement with beetroot juice could not improve the athletic performance among these 22‐year‐old male volunteers under the intermittent hypoxic training condition (Puype et al., 2015), which suggested that the nitrate in beetroot might only exert its effect under the aerobic condition. Under the condition of no oxygen or low oxygen, beetroot intake could not improve exercise performance and thereby lose the possibility of enhancing the endurance of anaerobic exercise. Apart from this, however, even under aerobic conditions, it was found that ingestion of beetroot supplementation did not work in all types of exercise. The acute ingestion of beetroot improved performance in a 30 s Wingate test with increased peak power and shortened time to reach this maximum power in resistance trained athletes (Wickham et al., 2019). However, it acted differently in recreational cyclists, national talent speed‐skaters, and Olympic‐level track cyclists that all were supplemented with 140 ml/d nitrate‐rich and nitrate‐depleted beetroot juice, respectively. When these subjects have undergone two 6‐day intakes, three 30‐s Wingate tests were carried out immediately. The results then showed in the tests that there was no difference of peak power over the Wingates between the subjects treated with nitrate‐rich and nitrate‐depleted beetroot, while nitrate‐rich beetroot treatment responding to time to peak power over the Wingates was improved by ∼2.8% compared with nitrate‐depleted treatment (Jonvik, Nyakayiru, et al., 2018). From these results, we speculate beetroot supplementation could enhance the capacity of accelerating during high‐intensity and sprint exercise. In the study of Lee J. Wylie et al., mean power output was found significantly increased in the nitrate‐rich group relative to the nitrate‐depleted group; however, such results could only present in high‐intensity intermittent exercise with the twenty four 6‐s all‐out sprints interspersed with 24 s of recovery (Wylie et al., 2016). The other evidence indicated that in severe‐intensity exercise, beetroot (nitrate) enhanced related exercise tolerance when the metabolic rate elevated (Breese et al., 2013). During a perfusion pressure challenge, supplementation with nitrate‐rich beetroot made it possible for restoring its exercise capacity (Bentley et al., 2017). Beetroot supplementation could also enhance the O_2_ delivery and thus extend the working hours before fatigue begins (Lee et al., 2015). In obese subjects, short‐term supplementation of beetroot could improve exercise tolerance (Rasica et al., 2018). For hill‐walkers, acute 24‐hr supplementation of nitrate in beetroot also had important implications for such exercise (Waldron et al., 2018). Even after blood donation, the intake of beetroot benefited to lower the cost of O_2_ and attenuate the decline in exercise performance (McDonagh et al., 2016). In spite of the above findings, there was no improvement in the exercise performance observed during the aerobic submaximal exercise despite nitric oxide increased following beetroot gel ingestion (Vasconcellos et al., 2017). Combination of beetroot (nitrate) and caffeine exhibited no obvious effect in athletic performance during submaximal or maximal treadmill running exercise (Oskarsson & McGawley, 2018). Even for intermittent exercise, ingesting nitrate in beetroot did not improve the performance of elite female athletes (Jonvik, van Dijk, et al., 2018). For moderately trained swimmers, it did not work at least for 100‐m and 200‐m freestyle swimming performance with beetroot intake (Esen et al., 2019). Thus, it is guessed that many factors involved in the limitation of beetroot‐induced effects.

At present, many studies are currently working on the inherent mechanisms by which beetroot enhances exercise performance. However, the insight on the relevant mechanism remains to be disputable, and all of the possible mechanisms described below are shown in Figure 3b. Roger A. Vaughan et al. held the point that nitrate‐containing beetroot extract was acted as an inducer to regulate the metabolic gene expression and mitochondrial content (Vaughan et al., 2015). Along with the up‐regulated oxidative metabolism, the expression of metabolic genes such as mitochondrial transcription factor A and peroxisome proliferator‐activated receptor gamma coactivator‐1 alpha was also elevated in C2C12 cells (Vaughan et al., 2015). In addition, during fatiguing resistance exercise for fourteen men, supplementation with beetroot‐based supplements could cause a neuromuscular advantage, that is, enhancing the neuromuscular efficiency (Flanagan et al., 2016). In the study of Scott Betteridge et al., however, they found that there was no significant decrease in muscle glycogen, phosphocreatine and increase in phosphorylated acetyl CoA carboxylase, muscle creatine and lactate with nitrate‐rich beetroot intake in males (Betteridge et al., 2016). A result revealed by J. Whitfield et al. that even under the action of beetroot, there existed no change in uncoupling proteins (UCP3, ANT1, ANT2), ADP sensitivity, P/O ratio, respiratory control ratios, and membrane potential. Therefore, the mitochondrial efficiency was found unaltered following beetroot supplementation in humans (Whitfield et al., 2015). As the results indicated the H_2_O_2_ emission increased after beetroot intake (Whitfield et al., 2015), it still required further study.

Along with possible effects on athletic performance, supplementation with beetroot was also likely to attenuate muscle soreness. Tom Clifford et al. found that when the level of pressure pain threshold (PPT) in a high and low dose of beetroot‐treated groups had returned to baseline at 72 hr postexercise, the level of which was still decreased in the placebo‐treated group (Clifford et al., 2016). In the subsequent study, they clarified that the phytonutrients in beetroot or the interaction between phytonutrients and nitrate might contribute to such effect (Clifford, Howatson, et al., 2017). Additionally, the benefits of beetroot were confirmed by the evidence that the beetroot did not adversely affect the body during exercise (Clifford, Bell, et al., 2017; Murphy et al., 2012). Due to these protective effects and enhanced performance activity, sports drinks based on beetroot are receiving increasing attention. For instance, Vanajakshi et al. made full use of lactobacillus to ferment beetroot and moringa to make a drink (Vanajakshi et al., 2015). A sports drink composed of beetroot extract, creatine, sulfuric acid, and calcium 3‐hydroxy‐3‐methylbutyrate was found to possess the capacity of increasing muscle blood flow, improving oxygen utilization, reducing exercise energy consumption, and thus to improve exercise performance. The application of beetroot products will be the research hotspot in the future.

### 2.5 Other activities

#### 2.5.1 Protective effect on liver and kidney

Beetroot is rich in antioxidant ingredients, and it would exert protective effect regarding oxidative stress in mice. Dimethylnitrosamine (NDEA) is an inducer to cause liver damage. After injection with NDEA in mice that were long‐term fed with beetroot juice, Krajka‐Kuzniak et al. found that beetroot juice prevented systemic liver damage through increasing the enzyme activity in phase I and phase II (Krajka‐Kuźniak et al., 2012; Vulić et al., 2013). After further exploring the mechanism for the liver protection and assessing the level of transaminase, transferase and glutathione S‐transferase in the normal hepatocytes, and liver tumor cells, the results indicated that beetroot protected the liver by activating Nrf2 and thus to exert antitumor activity. On radiation‐induced liver damage, it was found that beetroot treatment also significantly attenuated oxidative stress, induced cell apoptosis with caspase‐3 reduction, and besides decreased the activity of CYP450, enhanced GSH‐T in animals (Shedid et al., 2019). In addition to liver protection, beetroot also has a protective effect on the kidney. Co‐administration of beetroot ethanolic extract (BVEE) and gentamicin exhibited a reversal effect, that was, decreased the expression of TNF‐α, IL‐6, p65 in the nucleus and downregulated the activity of NF‐κB‐DNA binding, myeloperoxidase (MPO), nitric oxide in rats. The proteins link to apoptosis, such as Bcl‐2, Bax, and Caspase‐3 were also altered in the combination of BVEE and gentamicin (Iahtisham et al., 2019; Tesoriere et al., 2004). Taken together, these findings suggested that the reversal effect for beetroot against drug‐induced liver or kidney toxicity likely ascribed to its anti‐inflammatory, antioxidant, and anti‐apoptosis properties. These pathways were also later found to be associated with the beetroot in minimizing and preventing CPF neurotoxicity and testicular toxicity in rats (Albasher, Albrahim, et al., 2020; Albasher, Alsaleh, et al., 2020).

#### 2.5.2 Improving cognitive function

It has been recognized that the deficiency of nitric oxide was associated with cognition impairment. Beetroot has received increasing attention as a nitric oxide generator. In the research of healthy adults, Emma L Wightman et al. found that the altered parameters of prefrontal cortex cerebral blood flow (CBF) induced by nitrate‐containing beetroot were related to the cognitive function improvement (Wightman et al., 2015). Similarly, it has been found in the elderly that the supplementation of high nitrate in beetroot could increase regional CBF and thus enhance cognitive (Presley et al., 2011). In older adults, the improvement of femoral artery flow‐mediated dilatation (FMD) was also observed following supplementation (Walker et al., 2019). In addition, it was reported that following beetroot intake, the simple reaction time of patients with type 2 diabetes mellitus was significantly quicker compared with the placebo group, that was 327 ± 40 ms versus 341.8 ± 52.7 ms and *p* = .009 (Gilchrist et al., 2014). For the improvement in simple reaction time reach to 7 s was considered significant, the finding in beetroot application for the improvement of simple reaction time thus might be clinically significant. In HIV‐infected individuals with lower nitric oxide (NO) bioavailability and vascular dysfunction, beetroot juice ingestion acutely improved their vascular function similarly (Nogueira‐Soares et al., 2020). BRJ intervention also could provide important effect for those pregnancies associated with hypertension, endothelial dysfunction, and reduced NO bioavailability (Tropea et al., 2020). In spite of the findings, however, the observation in the improvement of cognitive function does not exist under any conditions. The research by Kevin G. Thompson et al. suggested that even the concentration of nitric oxide increased after beetroot consumption, there was no improvement in cognitive function before and after exercise in humans (Thompson et al., 2014). Therefore, it is important to study the adaptation population for making better use of beetroot to improve cognition.

#### 2.5.3 Others

**FIGURE 4:**
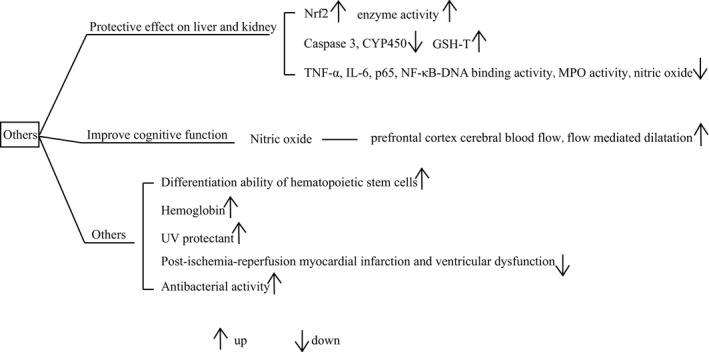
Diagram of other activity of beetroot. Nrf2, nuclear factor (erythroid‐derived 2)‐like 2; TNF‐α, tumor necrosis factor‐α; IL‐6, interleukin‐6; NF‐κB, nuclear factor‐kappa B; MPO, myeloperoxidase; UV, ultraviolet

Beetroot is currently confirmed to have other activities (Figure 4), which indeed expands its application despites there are few studies on these activities. For instance, Jinhee Cho et al. found that beetroot diet would improve mice survival rate that were exposed to the γ‐ray irradiation (Cho et al., 2017). The related mechanisms further were confirmed related to the promotion of cell proliferation and minimization of DNA damage in splenocytes and bone marrow. Moreover, it was observed that in beetroot‐treated mice, the differentiation ability of hematopoietic stem cells (HSCs) was up‐regulated. It was the combination of these altered factors that contributed to resisting ionizing radiation. Along with the differentiation of HSCs, the level of hemoglobin was also found to increase which had a link to the improvement of anemia in adolescent girls (Priya et al., 2013). Additionally, beetroot was found to act as a UV protectant (Born et al., 2015). Whether the beetroot possesses a corresponding protective effect on other types of radiation remains to be further studied.

Moreover, ingestion of beetroot provided protection against post‐ischemia‐ reperfusion (I/R) myocardial infarction and ventricular dysfunction in mice, which was found to be associated with the generation of endogenous H_2_S mediated by cystathionine‐g‐lyase (CSE) (Salloum et al., 2015). Studies have also found that beetroot could effectively increase the expression of Ca^2+^ in cardiomyocyte in mice, resulting in the improvement of ventricular contractile function (Pironti et al., 2016).

It was also found that beetroot could inhibit the infection with Escherichia coli, Pseudomonas aeruginosa, Citrobacter freundii, etc. in vitro, the function of which might be achieved by influencing the structure, function, and permeability of the microbial cells membrane (Vulić et al., 2013). Taking advantage of the antibacterial effect, beetroot could be applied to prevent dental caries by preventing acidification of human saliva (Hohensinn et al., 2016). Although beetroot has a broad spectrum of antibacterial activity, few studies have reported the mechanism for its antibacterial activity. The research of the specific molecular mechanism leading to the antibacterial effect would be the focus of future research. In these studies, however, the main components in beetroot playing the active effect have not been elaborated, which also will be the main content for future research.

## 3 CONCLUSION

**FIGURE 5:**
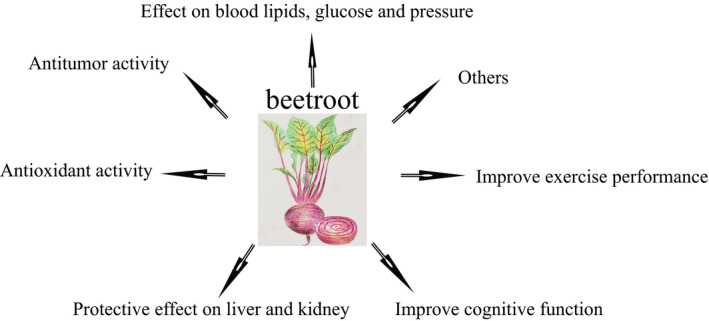
Summary of biological activity of beetroot

In this review, we have illustrated the ability of the main active beetroot ingredients in antioxidant, antitumor, lowing blood pressure, blood lipid, blood glucose, improving exercise performance, and many other aspects (Figure 5). Although not all of the active ingredients and its activities have been verified by clinical trials, related mechanisms have also not been fully elucidated; however, as a cheap and low‐toxic natural functional food, its application potential cannot be underestimated.

## CONFLICT OF INTEREST

The authors declare that they do not have any conflict of interest.

## ETHICAL APPROVAL

This study does not involve any human or animal testing.

## Data Availability

Data sharing is not applicable to this article as no datasets were generated or analyzed during the current study.
